# Systematic review of management for treatment-resistant depression in adolescents

**DOI:** 10.1186/s12888-014-0340-6

**Published:** 2014-11-30

**Authors:** Xinyu Zhou, Kurt D Michael, Yiyun Liu, Cinzia Del Giovane, Bin Qin, David Cohen, Salvatore Gentile, Peng Xie

**Affiliations:** Department of Neurology, the First Affiliated Hospital of Chongqing Medical University, 1 Yixueyuan Road, Yuzhong District, Chongqing, 400016 China; Institute of Neuroscience, Chongqing Medical University, Chongqing, China; Department of Psychology, Appalachian State University, Boone, North Carolina USA; Department of Clinical and Diagnostic Medicine and Public Health-Italian Cochrane Centre, University of Modena and Reggio Emilia, Modena, Italy; Department of Child and Adolescent Psychiatry, Université Pierre et Marie Curie, CNRS UMR 7222 “Institut des Systèmes Intelligents et Robotiques”, Hôpital de la Pitié-Salpétrière, Paris, France; Department of Mental Health, ASL Salerno, Italy

**Keywords:** Treatment-resistant depression, Adolescent, Systematic review

## Abstract

**Background:**

Current guidelines for treatment-resistant depression in adolescents remain inadequate. This study aimed to systematically review the management of treatment-resistant depression in adolescent patients.

**Methods:**

We conducted an electronic database search of PUBMED, EMBASE, Cochrane, Web of Science and PsycINFO for studies with adolescent treatment-resistant depression published up to January 2014. Treatment-resistant depression was defined as failure to respond to at least one course of psychological or pharmacological treatment for depression with an adequate dosage, duration, and appropriate compliance during the current illness episode. The Cochrane risk-of-bias method was used to assess the quality of randomized controlled trials. A meta-analysis of all active treatments was conducted.

**Results:**

Eight studies with 411 depressed adolescents that fit predetermined criteria investigated pharmacological treatments and psychotherapies. Six were open-label studies, and two were randomized controlled trials. The overall response rate for all active treatments investigated was 46% (95% CI 33 to 59; N = 411) with a moderately high degree of heterogeneity (I^2^ = 76.1%, 95% CI = 47%-86%). When only the two randomized trials were included, the overall response rate of active treatment was 53% (95% CI = 38-67; N = 347). In these randomized trials, SSRI therapy plus CBT was significantly more effective than SSRI therapy alone, while amitriptyline was not more effective than placebo.

**Conclusions:**

Approximately half of the adolescents who presented with treatment-refractory depression responded to active treatment, which suggests that practitioners should remain persistent in managing these challenging cases. The combination of antidepressant medication and psychotherapy should be recommended for adolescents who present with treatment-resistant depression.

**Electronic supplementary material:**

The online version of this article (doi:10.1186/s12888-014-0340-6) contains supplementary material, which is available to authorized users.

## Background

Depression in adolescents is a major public health problem with an estimated point prevalence of about 2-5% among teens between 13 and 18 years of age. Approximately 20% of adolescents experience at least one episode of major depression before adulthood [1,2]. Despite advances in the treatment of depression in adolescents (e.g., medication, psychotherapy), it is estimated that 30% to 40% of patients do not show an adequate clinical response to the initial treatment when defined as at least a 50% reduction in symptoms. Fava M, 2003 [3,4] Adolescents who do not respond to an adequate initial treatment dosage, termed treatment-resistant depression (TRD), have a high likelihood of recurrence into adulthood [5], and compared with these non-refractory patients, display higher suicide rates, more serious impairments in social functioning, worse school achievement, and more relational problems with family members and peers [6-8].

Many studies have been conducted in the field of adult depression, and there are some similarities between adolescent and adult depression; for example, cognitive-behavioral therapy (CBT) and selective serotonin reuptake inhibitors (SSRIs) are routinely used to treat both adults and adolescents [9,10]. In contrast, several studies have substantiated differences between these two populations; for example, there are higher odds of a family history of psychopathology and developmental problems in adolescents [11,12]. For the management of adult TRD, two main strategies have been proposed -- switching to another therapeutic class or augmentation. These two strategies are generally considered to be relevant to different populations -- non-responders and partial responders, respectively [13,14]. Partial or non-responses to conventional treatments for depression may be moderated or mediated by the presence of stressors such as losses, abuse, neglect, and ongoing conflicts and frustrations. Moreover, the effects of these stressors also depend on the adolescents’ negative attributional styles for interpreting and coping with stress, available support systems, and genetic factors [15]. Other factors -- such as the presence of comorbid disorders (e.g., anxiety, substance abuse, ADHD, eating disorders), medical illness, medication use/abuse, biological factors, and sociocultural factors -- have also been associated with the development and maintenance of depressive symptomatology [16-18].

Furthermore, given that adolescents typically show a lower and potentially slower response rate to antidepressants as compared to adults, there is ample justification to analyze the management of depressed adolescents separately from adults [19-21]. Unfortunately, there is a paucity of data on the clinical management of adolescent patients with TRD. A previous review of TRD in adolescents examined a total of six studies (i.e., five open-label trials and one randomized controlled trial) [22]. However, the review was not comprehensive or systematic and the generalizability of the findings was limited.

In light of the prevalence and adverse consequences of adolescent TRD (e.g., suicide), the importance of having updated clinical data on the management of TRD in teens cannot be overstated. However, an up-to-date, systematic review on the management of TRD in adolescents has not been performed. Therefore, in this review, we conducted a meta-analytic review of the efficacy of pharmacological and psychosocial interventions for adolescent TRD.

## Methods

### Search strategy

We searched five electronic databases (PubMed, Embase, the Cochrane Library, Web of Science, and PsycINFO) from inception to January 2014 with Medical Subject Headings (MeSH) and text words. Additional file 1: Table S1 includes a detailed systematic search strategy. We also reviewed the clinical trial registry (clinicaltrials.gov), the websites of pharmaceutical companies, and relevant reports from the U.S. Food and Drug Administration (FDA) website. No language restrictions were imposed on the searches. Additional studies were obtained by scanning reference lists of relevant reviews and initially eligible trials.

### Selection criteria

We included all primary research evaluating pharmacological or psychological for adolescent TRD. The participants were both boys and girls (aged less than 18 years) with a primary diagnosis of unipolar major depressive disorder according to the standardized diagnostic criteria set forth in the *Diagnostic and Statistical Manual of Mental Disorders* (*DSM-III* [23], *DSM-III-R* [24], *DSM-IV* [25]). We defined TRD as those who failed to respond to at least one psychological or pharmacological treatment for depression with an adequate dosage, duration, and appropriate compliance for the particular episode [3]. We excluded trials with duplicate secondary analyses, bipolar depression, single case reports, physical treatments, or studies lacking useable data.

### Outcome measures

We defined outcomes dichotomously as the proportion of patients who responded to the treatment (or placebo or control where appropriate). Response was defined as a reduction of at least 50% in the score of depression rating scales [26], such as Children Depression Rating Scale-Revised (CDRS-R) [27] and Hamilton Depression Rating Scale (HAMD) [28], or rating as “1” (very much improved) or “2” (much improved) in the Clinical Global Impression-Improvement Scale (CGI-I) [29].

### Data extraction

Two authors (BQ and YYL) independently reviewed the titles and abstracts, updated the search by reviewing references, identified full-text articles by the same eligibility criteria, and completed a standardized data extraction form. Any disagreements were resolved by another review author (XYZ). We also assessed the methodological quality of randomized controlled studies (RCTs) using the risk of bias assessment tool from the Cochrane Handbook [30].

### Statistical analysis

We performed a meta-analysis of all active treatments and used the StatsDirect software package (version 2.8.0, Cheshire, UK) to analyze the data. We calculated the I^2^ statistic, which estimates the percentage of variation across studies that is due to heterogeneity rather than chance [31]. We decided to use a random-effects model since there was expected clinical diversity in these different treatments. We performed a funnel plot to examine publication bias and computed the Egger statistic as an indicator of bias [32]. The overall effect sizes were calculated based on the pooled proportions and 95% confidence intervals (CIs) for any given treatment. Moreover, we also calculated the pooled proportions and 95% CIs for any treatment reported by at least two studies separately. We performed a sensitivity analysis that only included randomized controlled trials. We also conducted a subgroup analysis based on the type of refractory antidepressant and another subgroup analysis based on augmentation therapy and switching therapy.

## Results

**Figure 1:**
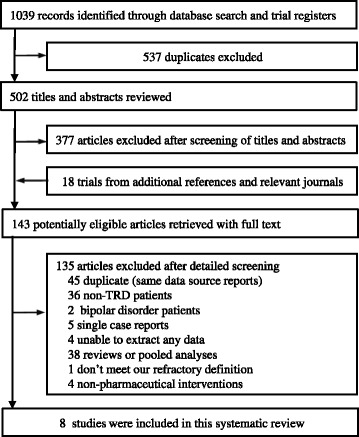
**Literature search.**

A total of 1039 records were identified through the initial database search. After excluding 537 duplicate records, we retrieved 502 potentially relevant studies. Of these, 377 articles were excluded because or irrelevant titles and abstracts, and 18 additional articles were identified as potentially relevant from the references of these trials and relevant reviews. Then, 135 citations were excluded after two reviewers independently read the full texts (BQ and YYL). Among these, we also excluded one trial because its definition of TRD did not meet our criterion [33]. Finally, eight studies including 411 patients [34-41] met all our inclusion criteria and were included in the systematic review (Figure 1).

### Description of included studies

The Table 1 summarizes the characteristics and outcomes of each trial. The trials were published between 1988 and 2011. The sample size ranged from 5 to 334 patients, with a mean sample size of 51 per trial, and the mean age of participants was 15.9 years (range 12–18 years). Six studies defined TRD as failure to respond to one antidepressant, and two studies defined TRD failure to respond to at least two antidepressants and/or psychotherapies. Most trials recruited convenience participants not designed to be epidemiologically representative of an underlying population. Only two studies provided information about patients who were referred but did not participate, reporting about one-tenth of those potentially eligible participated (33/380 and 334/3258). All studies, with the exception of the one including only females, involved both female and male patients, and the overall female-to0male ratio was approximately 2.4:1.

**Table 1:** **Baseline characteristics and outcomes of included studies**

**Study**	**Age (years)**	**Diagnostic criteria**	**Baseline score**	**Female (%)**	**TRD definition**	**Trial length (weeks)**	**Treatment**	**Response Definition**	**Measure outcome**
Birmaher [34]^*^	12-18	DSM-III-R	HAMD ≥15	70	Failure to response to at least two antidepressants	10	Amitriptyline, 50–300 mg/d (N = 13); placebo (N = 14)	≥50% reduction in HAMD score	Amitriptyline: 10/13; placebo: 11/14
Boulos [35]	14-18	DSM-III-R	HAMD ≥17	57	Failure to respond to at least two consecutive months with a TCA	6-7	Fluoxetine, 5–40 mg/d (N = 7)	≥50% reduction in HAMD score	Fluoxetine: 2/5
Brent [36]^*^ (TORDIA)	12-18	DSM-IV	CDRS-R ≥ 40	71	Failure to response to an SSRI regimen for at least eight weeks	12	A second SSRI (N = 85); venlafaxine (N = 83); a second SSRI plus CBT (N = 83); venlafaxine plus CBT (N = 83)	≥50% reduction in CDRS-R score	No CBT: CBT = 68/168:91/166
Ghaziuddin [37]	15-18	DSM III-R	HAMD ≥11	67	Failure to response to an adequate trial of a TCA for at least four weeks	4-16	Fluoxetine, 20–60 mg/d (N = 6)	≥50% reduction in HAMD score	Fluoxetine: 2/6
Kondo [38]	13-18	DSM-IV	CDRS-R ≥40	100	Failure to response to fluoxetine treatment for over eight weeks	8	Augmentation with creatine, 4 g/d (N = 5)	≥50% reduction in CDRS-R score	Creatine: 3/5
Pathak [39]	13-18	DSM-IV-TR	—	60	Failure to respond to at least an eight-week trial of an SSRI with an adequate dose	Case series (4–16)	Augmentation with quetiapine, 150–800 mg/d (N = 10)	CGI-I of 1 or 2	Quetiapine: 7/10
Ryan [40]	14-19	DSM III	—	79	Failure to respond to at least a four-week trial of a TCA	Case series (3–16)	Augmentation with lithium, 600–1500 mg/d (N = 14)	CGI-I of 1 or 2	Lithium: 6/14
Strober [41]	13-18	DSM III-R	HAMD-21 ≥ 16	71	Failure to response to imipramine at least six weeks	3	Augmentation with lithium, 900 mg/d (N = 24)	≥50% reduction in HAMD score	Lithium: 2/24

*Abbreviations:* *DSM-III DSM-III-R DSM-IV DSM-IV-TR* Diagnostic and Statistical Manual of Mental Disorders III version, III revision version, IV version, IV text revision version, *CDRS-R* Children Depression Rating Scale-Revised, *CGI-S* Clinical Global Impressions Severity Subscale, *HAMD* Hamilton Depression Rating Scale, *TRD* treatment-resistant depression, *SSRI* selective serotonin reuptake inhibitor, *TCA* tricyclic antidepressant, *CBT* cognitive behavior therapy, *CGI-I* Clinical Global Impressions Improvement Subscale.

*Randomized controlled trial (RCT).

### Quality of literature

**Figure 2:**
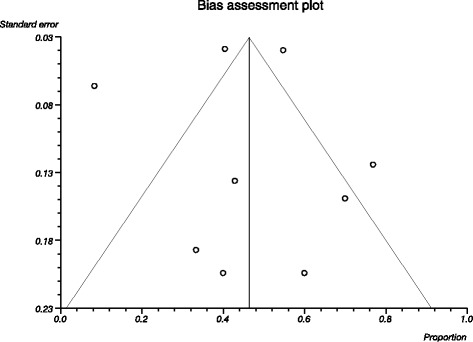
**Funnel plot of the included studies examining publication bias.** *There was no significant asymmetry detected, indicating that no publication bias was present.

Of the eight included studies, only two studies were RCTs, which were rated as being of high-quality. The other six studies were described as open-label studies, which were judged to be of lower-quality. Additional file 2: Figure S1 shows the quality of these two RCTs based on the Cochrane risk-of-bias method. The overall quality of two RCT studies was rated as good, and most question-based entries in trials met criteria for low risk of bias. About the TORDIA study, we regarded the other bias as high risk, because they changed the treatment options from paroxetine to citalopram. We visually inspected the inverted funnel plots of these eight studies, which appeared to be approximately symmetrical (Figure 2). Because the total number of studies was too small to show clear asymmetry, we performed the Egger test, and the results showed that the depression outcomes (t = 0.34, *P* = 0.83) were not influenced by publication bias.

### Efficacy of treatments

**Figure 3:**
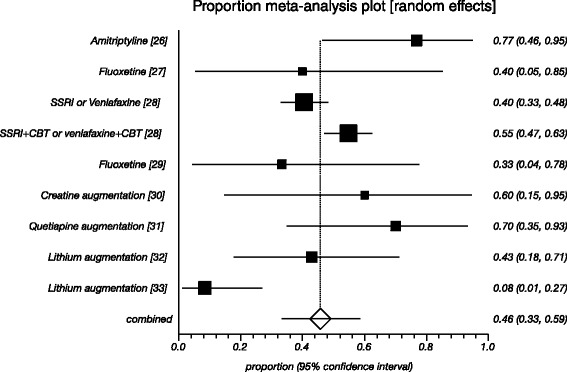
**Proportional meta-analysis of the included studies with weighted response rates and 95% confidence intervals.**

The overall response rate for the nine active treatments (one RCT included two active treatments) investigated was 46% (95% CI = 33 59, N = 441; Figure 3). The I^2^ statistic was 76.1% (95% CI = 47%-86%), indicating a moderately high degree of heterogeneity between the studies. When we included only randomized trials, the overall response rate was 53% (95% CI = 38-67, N = 347; Additional file 3: Figure S2). However, in the placebo controlled trial [26], there was no significant difference in response rates between active treatment (76.9%) and placebo (78.6%). In the subgroup-analysis for the type of refractory antidepressant (Additional file 4: Figure S3), the overall response rate of TCA-resistant TRD was 29% (95% CI = 11-51, N = 49), and the overall response rate of SSRI-resistant TRD was 51% (95% CI = 39-63, N = 349). In the 13 patients with multi-drug-resistant TRD, the overall response rate was 75% (95% CI = 50-93, N = 13). In this review, the treatments for which there was evidence from at least two studies (Additional file 5: Figure S4) were SSRI therapy (38%, 95% CI = 15-65, N = 11) and lithium augmentation (24%, 95% CI = 1-61, N = 38). In the other subgroup-analysis, the overall response rate of switching therapy was 49% (95% CI = 30-67, N = 53), and the overall response rate of augmentation therapy was 42% (95% CI = 14-73, N = 192) (Additional file 6: Figure S5).

### Randomized controlled trials

The TORDIA trial [36] evaluated the effects of four active treatment groups, including switching to a second SSRI (paroxetine, citalopram, or fluoxetine, 20–40 mg/d), switching to venlafaxine (150–225 mg/d), switching to a different SSRI plus cognitive behavioral therapy (CBT), or switching to venlafaxine plus CBT, in 334 adolescents with SSRI-resistant TRD. Randomization was assigned to one of four treatment regimens in a 2 × 2 factorial design. The intent was for study participants, clinicians, and independent evaluators to be blinded to medication treatment assignment and for independent evaluators to be blinded to CBT assignment. The CGI-I, the CDRS-R, Beck Depression Inventory (BDI), Mania Rating Scale (MRS), and Side Effects Form for Children and Adolescents were evaluated at baseline and at six weeks and twelve weeks. The CBT plus a switch to either medication regimen showed a higher response rate (54.8%) than a medication switch alone (40.5%), but there was no significant difference in response rate between venlafaxine and a second SSRI (48.2% versus 47.0%). Moreover, there was a greater increase in the diastolic blood pressure and pulse rate and more frequent occurrence of skin problems during venlafaxine therapy relative to SSRI therapy. Across all four groups, three out of ten (30.8%) participants withdrew.

Birmaher et al. [34] compared amitriptyline (50–300 mg/d) to placebo in 27 depressed adolescents. Randomization was balanced to match approximately for age (≥15 years versus <15 years) and sex. Clinicians did not adjust the dosage based on response and tolerance. Participants and clinicians were blinded to medication treatment assignment. All patients were evaluated by the HAMD, CGI-I, BDI, a side effects checklist, and Children’s Global Assessment Scale (CGAS) on a weekly basis during the ten-week treatment. Interestingly, no statistically significant differences were found between amitriptyline and placebo in these outcomes at the point of randomization and at the end of treatment. Both treatments were well-tolerated, and the side effects were mildly elevated in the amitriptyline group.

### Open-label studies on antidepressant medications

Fluoxetine was reported in two non-randomized trial. Boulos et al. [35] reported the effectiveness of fluoxetine (5–40 mg/day) in an open naturalistic trial. Six of the seven adolescent patients (85.7%) completed at least six consecutive weeks of treatment, and one suffered a rash and withdrew. Ghaziuddin et al. [37] investigated fluoxetine (20–60 mg/day) in an open cross-over trial with six hospitalized adolescents. Fluoxetine was well-tolerated, and discontinuation was not necessary in any subject.

Two open studies of tricyclic antidepressant (TCA) refractory depression investigating lithium augmentation had an identical design. Ryan et al. [40] reported a retrospective chart review of 14 adolescents. Six patients achieved a good response, and no case necessitated its discontinuation due to side effects from lithium augmentation. Strober et al. [41] conducted a three-week lithium augmentation trial in 24 adolescents. Lithium was started at 900 mg and then increased by clinical response. The addition of lithium was associated with relatively few side effects.

Pathak et al. [39] augmented antidepressant therapy with quetiapine (150–800 mg/day, median = 200 mg/day), in 10 adolescent patients. Doses of pre-existing antidepressants remained unchanged during the period of evaluation. Side effects included sedation and weight, and there was no serious adverse event. Kondo et al. [38] conducted an open trial of adjunctive creatine with fluoxetine. Five female adolescents were treated with 4 g/day creatine by mouth for eight weeks. Adverse events were self-limited with no unresolved treatment-emergent side effects.

## Discussion

There is a paucity of systematic evidence to guide practitioners in the management of adolescent TRD despite the high morbidity and severe impairment in these young patients. In this review, we found eight studies of pharmacotherapy or the combination of medication and psychotherapy that met our inclusion criteria. Our findings indicated that half of adolescents with refractory depression responded adequately and that most treatments were well-tolerated. One high-quality study suggested that a combination of a SSRI/SNRI antidepressant and CBT was significantly more effective than antidepressant therapy alone [36]. The findings are similar to the more definitive adult study of treatment resistant depression (STAR*D) [42].

The current treatment guidelines for the management of depression in adolescents always start with psychological education and supportive management followed by the addition of psychological therapy [12,15,43-45]. Cognitive behavioral therapy (CBT) or interpersonal psychotherapy (IPT) should be considered as first-line treatment for adolescents with depressive symptoms and mild to moderate depression. Pharmacological treatment (e.g. SSRIs) should be considered for acute, short-term reduction of depressive symptoms in adolescents with moderate to severe MDD [44,45]. CBT may be added to/continued with SSRI therapy in order to reduce the risk of suicidal ideation and improve functioning in adolescents with severe MDD [43]. However, there are still a considerable number of adolescents which have not achieved the response or remission with depression despite receiving the standard treatment recommended by these guidelines. Unfortunately, the paucity of studies on how to manage TRD in adolescents reveals a gap between the current knowledge base and the need for evidence-based data to guide clinical care.

According to the findings from TORDIA, switching to an antidepressant (SSRI or SNRI) in combination with CBT was superior (54.8%) to switching to medication alone (40.5%). These results are generally consistent with a previous Cochrane meta-analysis that showed combination therapy (65.9%) to be more effective than antidepressant medication alone (57.8%), yet the benefit from combination therapy did not reveal a significant difference (OR = 1.56, 95% CI, 0.99-2.27) [46]. However, it should be noted in the TORDIA trial [36], the medication-only condition actually had “clinical management” sessions during the acute phase that might have had active “psychotherapy-like” components (e.g., encouragement, assessment). Moreover, the CBT in the acute phase of TORDIA trial was relatively weak for such a severe variant of depression (i.e., average: 8.3 sessions). Thus, although these results show a modest benefit from combination therapy, the dosages of psychotherapy needed for adolescent TRD should be further studied [12,47].

In another RCT that was judged to be of higher-quality [34], amitriptyline did not appear to be significantly efficacious for adolescents with severe TRD despite the high response rate to TCA therapy. The high response rate of amitriptyline may have been associated with other features of the study (e.g., intensive inpatient, milieu therapy). A previous RCT of amitriptyline for the treatment of adolescents with MDD revealed similar findings regarding the efficacy of amitriptyline [48]. Due to the limited clinical benefits and significant side effects associated with TCA use, TCA drugs should not be recommended for use in the management of adolescent TRD [38].

All statements on the efficacy of treatments must be tempered by the potential biases and uncertainties that result from the choice of patients and therapies. In our analysis, those patients with multi-drug-resistant TRD showed a high response rate, and patients with SSRI-resistant TRD appeared to display a higher response rate than those with TCA-resistant TRD.

### Limitations

Several limitations to this systematic review should be noted. First, the summary response rates were derived primarily from open-label studies and only two RCTs, so they should be considered in light of these evolving findings. Second, although we chose the minimum criteria from the NICE Clinical Guidelines, there are different definitions of treatment resistance in adolescent TRD. National Collaborating Centre for Mental Health (NCCMH), 2005 [12] Our treatment refractory criteria were inclusive to ensure that we considered a broad range of evidence, but consequently there was a high degree of heterogeneity in the included studies. Third, trial durations ranged from three weeks to sixteen weeks, and few studies were less than six weeks in duration. Thus, the relatively short duration in the included studies may have led to an underestimation of treatment efficacy.

## Conclusions

Among adolescent depressed patients, the failure to respond to conventional treatment is common, yet there is a paucity of data on which evidence-based treatment decisions can be made. In this systematic review of TRD adolescents who had failed to respond to at least one antidepressant or combination psychotherapy, half of patients responded adequately to active treatment. These findings suggest that TRD in adolescents requires more patience, persistence, and systematic effort than adolescent MDD. The TORDIA trial suggests that an antidepressant (SSRI/SNRI) plus CBT has a significant additional benefit over antidepressant therapy alone in refractory adolescents. However, high-quality RCTs comparing therapies for TRD in adolescents with a more intensive array of psychotherapy dosages are needed to improve the evidence base for this debilitating illness. Also, it is emphasized that not only are there a paucity of studies on how best to manage TRD, the current guidelines and the implicit sequence of care embedded within them are not empirically based. Our field would benefit from studies that directly evaluate sequences of care.

## Additional files

Additional file 1: Table S1. Results from the Systematic Search Strategy*. Additional file 2: Figure S1. Risk of bias analysis. Detailed risk of bias analysis across randomized controlled trials. Additional file 3: Figure S2. Proportion meta-analysis for Randomized Controlled Trials. Additional file 4: Figure S3. Proportion meta-analysis for subgroup analysis of the type of refractory antidepressants. Additional file 5: Figure S4. Proportion meta-analysis for treatments reported by at least two studies. Additional file 6: Figure S5. Proportion meta-analysis for subgroup analysis of the type of sequential therapy.
