# Supplementary material for: Systematic review of management for treatment-resistant depression in adolescents
Source: BMC Psychiatry. 2014 Nov 30;14:340. doi: 10.1186/s12888-014-0340-6 (PMC4254264; doi:10.1186/s12888-014-0340-6)
Supplement: Additional file 1: Table S1. — Results from the Systematic Search Strategy*. [file 12888_2014_340_MOESM1_ESM.doc]

**Additional file 1: Table S**1. Results from the Systematic Search Strategy*

| **Databases:** | **Citations** |
| --- | --- |
| Pubmed | 41 |
| Cochrane | 226 |
| Web of Science | 268 |
| Embase | 202 |
| PsycInfo | 101 |
| ***Total (*databases)** | ***838*** |
| **Trial registers:** USA (clinicaltrials.gov) | 201 |
| ***Total*** | ***1039*** |

*Explicit search strategy: title / abstract = (depress*) AND title / abstract =( child* or teenager* or teen* or youth* or adolescen* or pediatric* or prepubertal or juvenile ) AND title / abstract =(resist* or refract* or non-respon* or nonrespon* or ‘inadequate respon*’ or ‘partial or respon*’ or ‘insufficient respon*’). Additional relevant studies were obtained by scanning relevant systematic reviews, meta-analyses, and reviews as well as reference lists of eligible trials.
