# Supplementary material for: Systematic review of management for treatment-resistant depression in adolescents
Source: BMC Psychiatry. 2014 Nov 30;14:340. doi: 10.1186/s12888-014-0340-6 (PMC4254264; doi:10.1186/s12888-014-0340-6)
Supplement: Additional file 2: Figure S1. — Risk of bias analysis. Detailed risk of bias analysis across randomized controlled trials. [file 12888_2014_340_MOESM2_ESM.pdf]

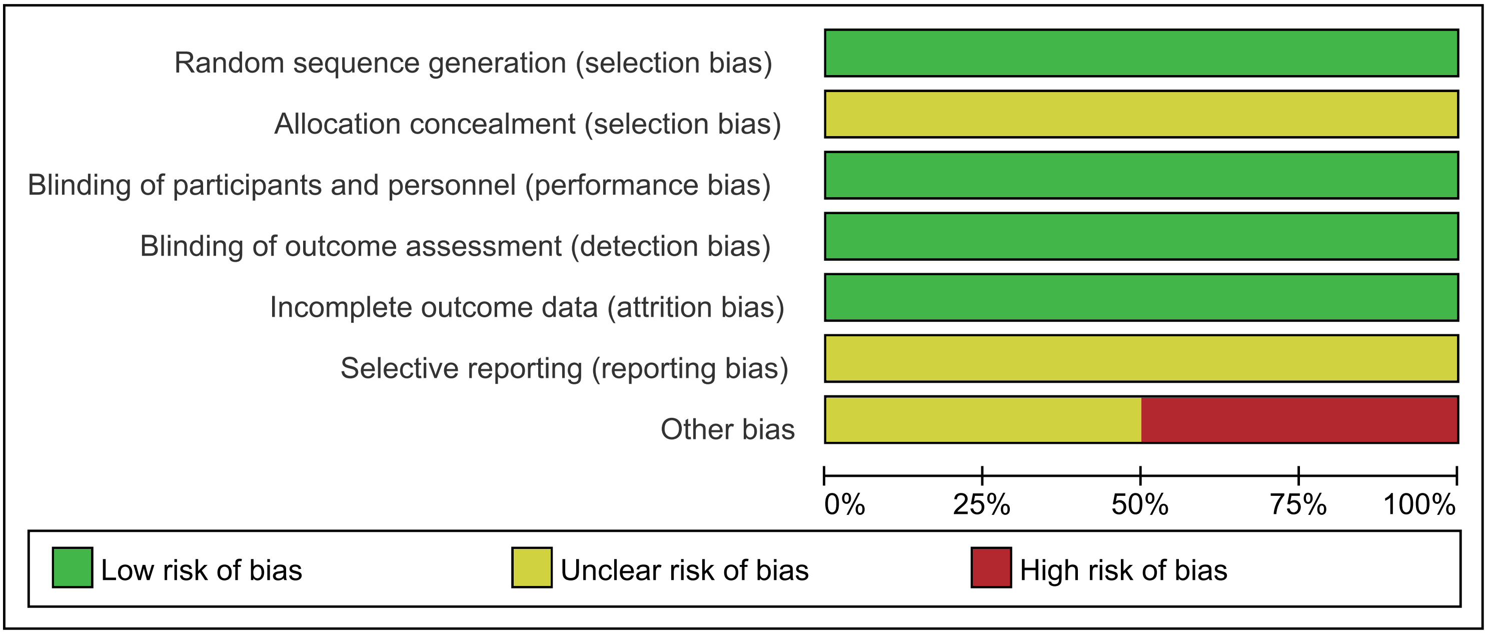
Random sequence generation (selection bias)
Allocation concealment (selection bias)
Blinding of participants and personnel (performance bias)
Blinding of outcome assessment (detection bias)
Incomplete outcome data (attrition bias)
Selective reporting (reporting bias)
Other bias
0%
25%
50%
75%
100%
Low risk of bias
Unclear risk of bias
High risk of bias

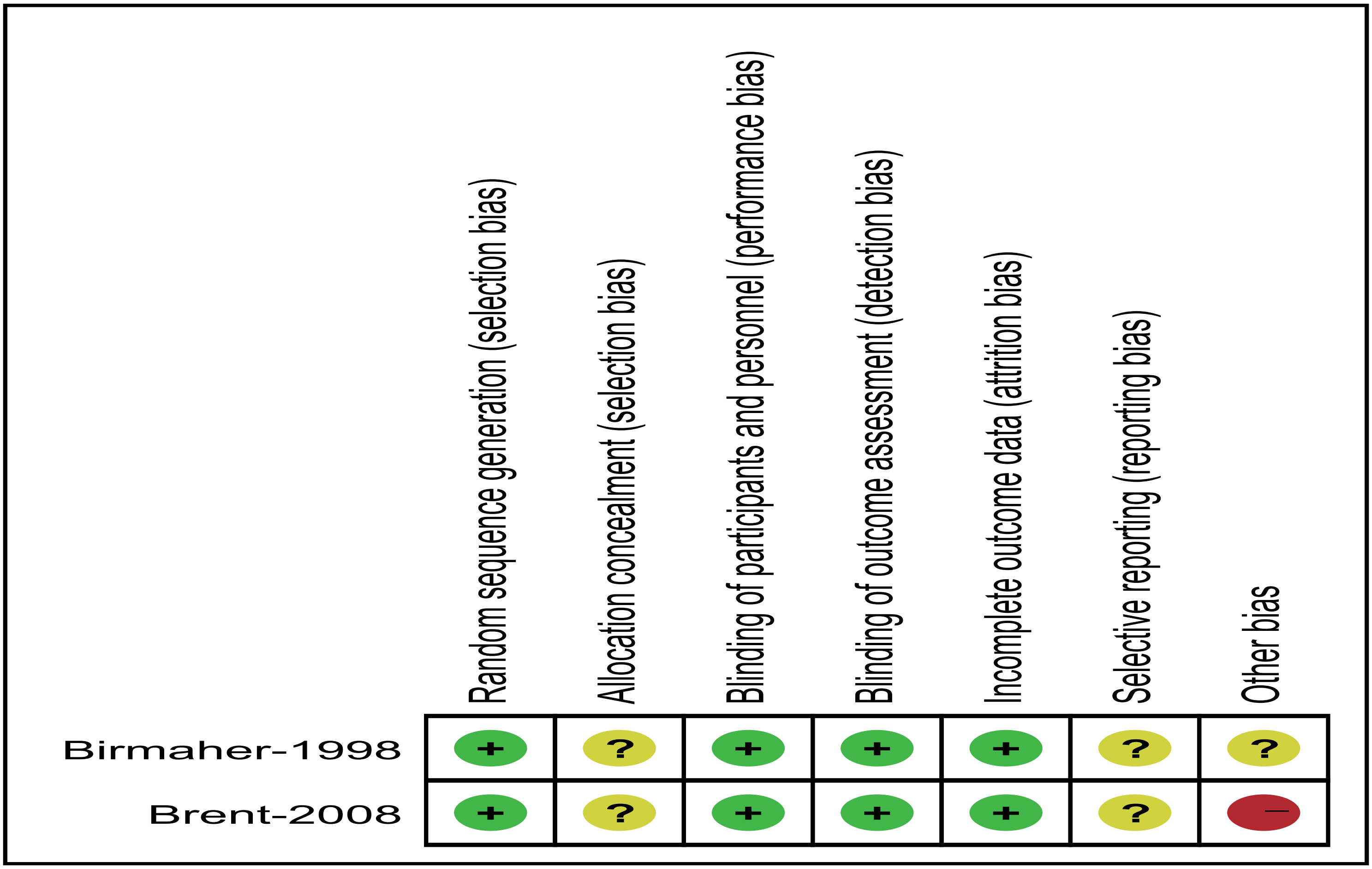
Random sequence generation (selection bias)
Allocation concealment (selection bias)
Blinding of participants and personnel (performance bias)
Blinding of outcome assessment (detection bias)
Incomplete outcome data (attrition bias)
Selective reporting (reporting bias)
Other bias
Birmaher-1998
Brent-2008
+
?
–
