# Supplementary figures and images for: Systematic review of management for treatment-resistant depression in adolescents
Source: BMC Psychiatry. 2014 Nov 30;14:340. doi: 10.1186/s12888-014-0340-6 (PMC4254264; doi:10.1186/s12888-014-0340-6)

Proportion meta-analysis plot [random effects]

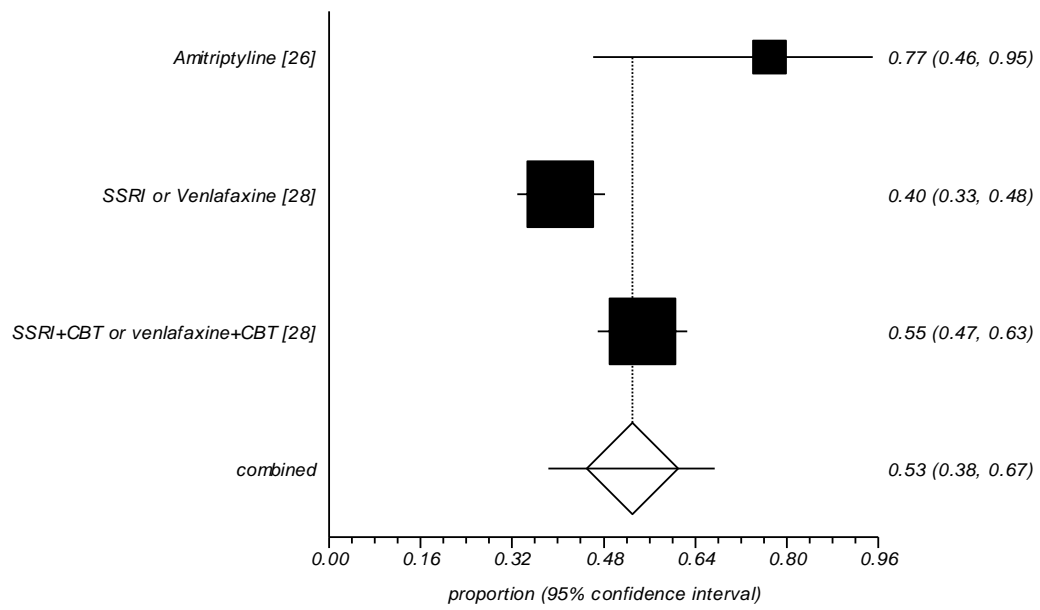

Supplement: Additional file 3: Figure S2. — Proportion meta-analysis for Randomized Controlled Trials. [file 12888_2014_340_MOESM3_ESM.pdf]

A

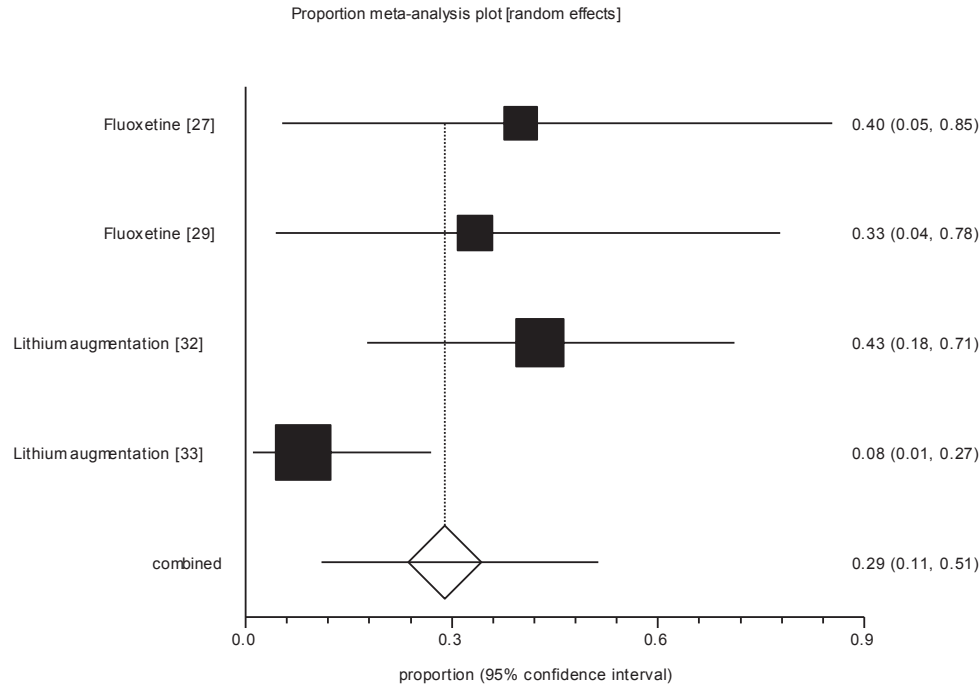

B

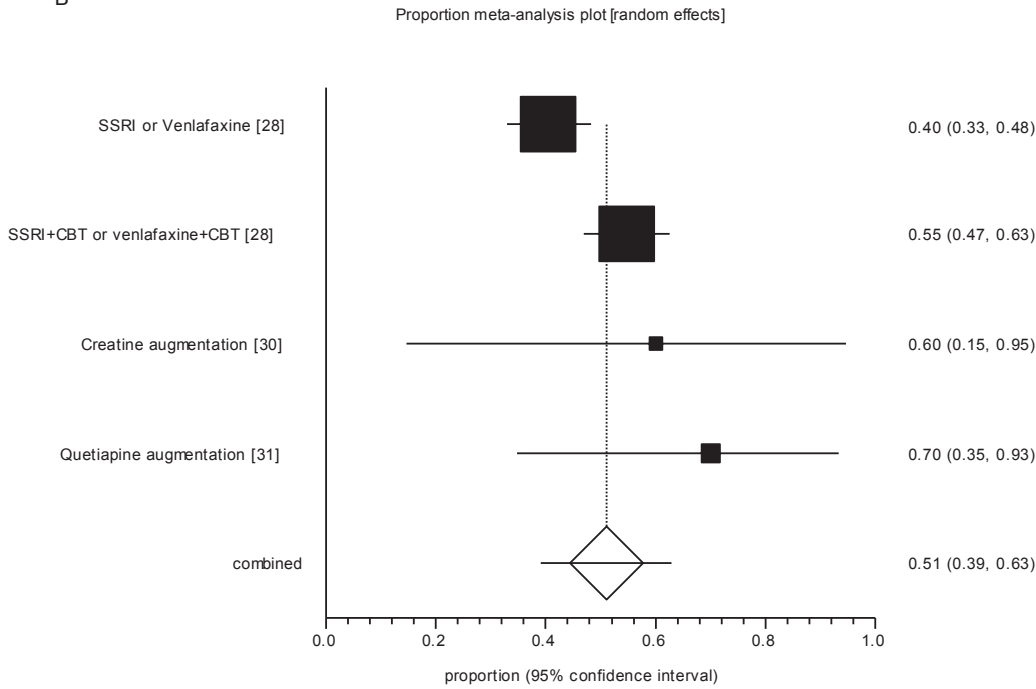

C

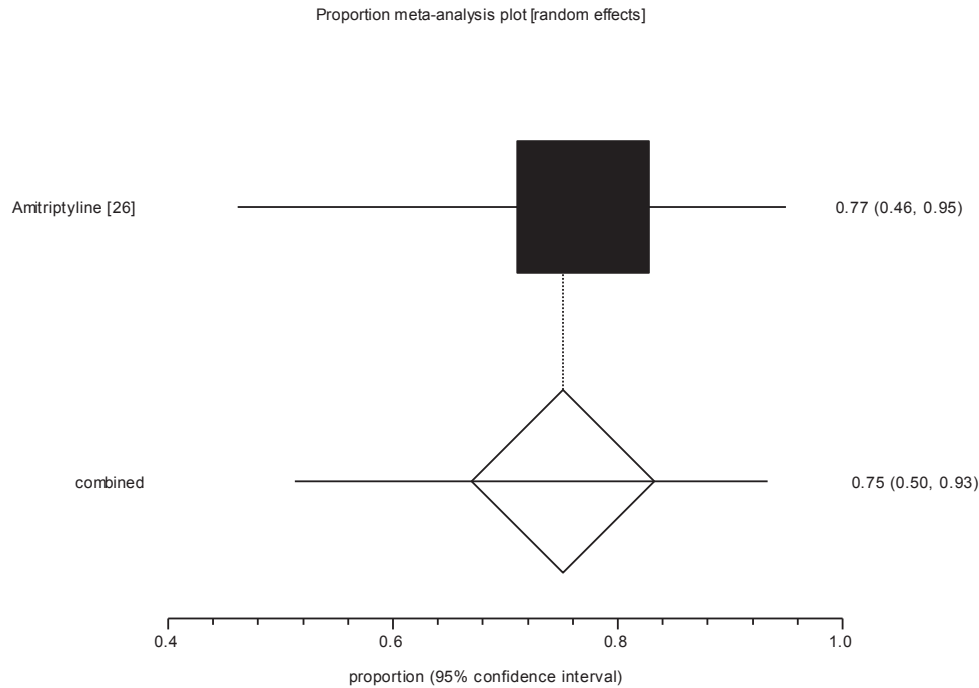

Supplement: Additional file 4: Figure S3. — Proportion meta-analysis for subgroup analysis of the type of refractory antidepressants. [file 12888_2014_340_MOESM4_ESM.pdf]
