# Supplementary material for: Systematic review of management for treatment-resistant depression in adolescents
Source: BMC Psychiatry. 2014 Nov 30;14:340. doi: 10.1186/s12888-014-0340-6 (PMC4254264; doi:10.1186/s12888-014-0340-6)
Supplement: Additional file 5: Figure S4. — Proportion meta-analysis for treatments reported by at least two studies. [file 12888_2014_340_MOESM5_ESM.pdf]

A

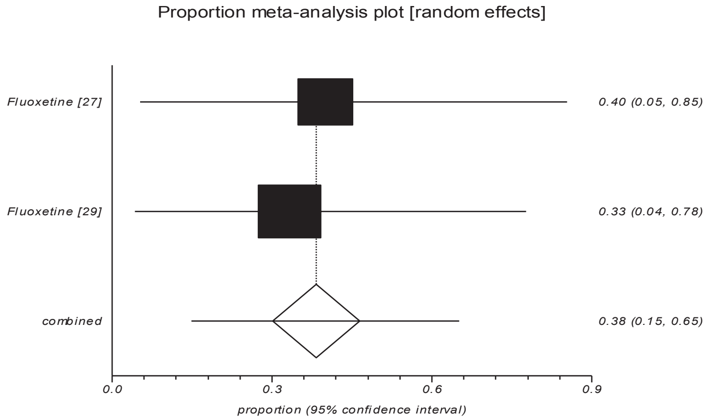
Proportion meta-analysis plot [random effects]
Fluoxetine [27]
Fluoxetine [29]
combined
0.40 (0.05, 0.85)
0.33 (0.04, 0.78)
0.38 (0.15, 0.65)
0.0
0.3
0.6
0.9
proportion (95% confidence interval)

B

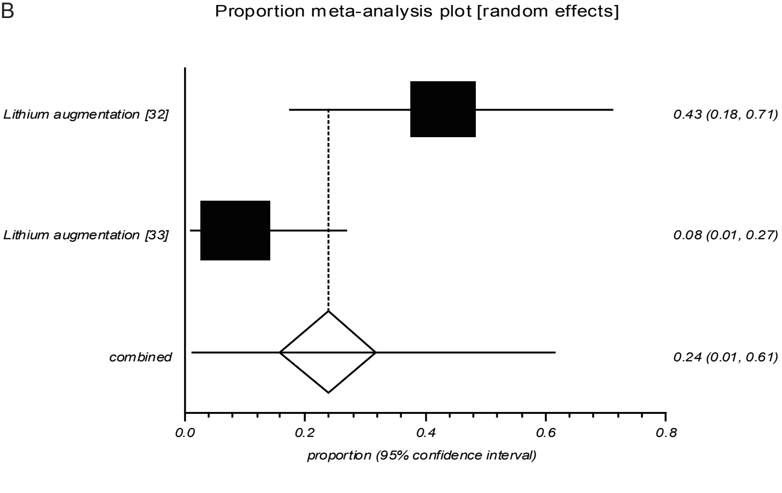
Proportion meta-analysis plot [random effects]
Lithium augmentation [32]
Lithium augmentation [33]
combined
0.43 (0.18, 0.71)
0.08 (0.01, 0.27)
0.24 (0.01, 0.61)
0.0
0.2
0.4
0.6
0.8
proportion (95% confidence interval)
