# Supplementary material for: Systematic review of management for treatment-resistant depression in adolescents
Source: BMC Psychiatry. 2014 Nov 30;14:340. doi: 10.1186/s12888-014-0340-6 (PMC4254264; doi:10.1186/s12888-014-0340-6)
Supplement: Additional file 6: Figure S5. — Proportion meta-analysis for subgroup analysis of the type of sequential therapy. [file 12888_2014_340_MOESM6_ESM.pdf]

A

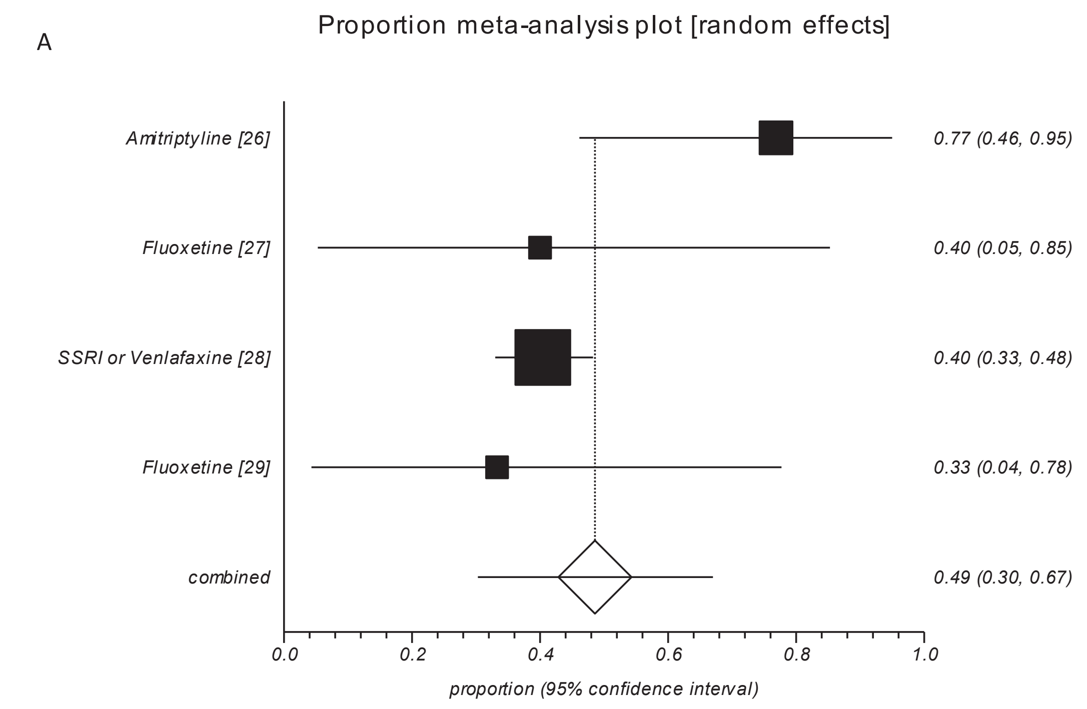

Proportion meta-analysis plot [random effects]
Amitriptyline [26]
Fluoxetine [27]
SSRI or Venlafaxine [28]
Fluoxetine [29]
combined
0.77 (0.46, 0.95)
0.40 (0.05, 0.85)
0.40 (0.33, 0.48)
0.33 (0.04, 0.78)
0.49 (0.30, 0.67)
0.0
0.2
0.4
0.6
0.8
1.0
proportion (95% confidence interval)

B

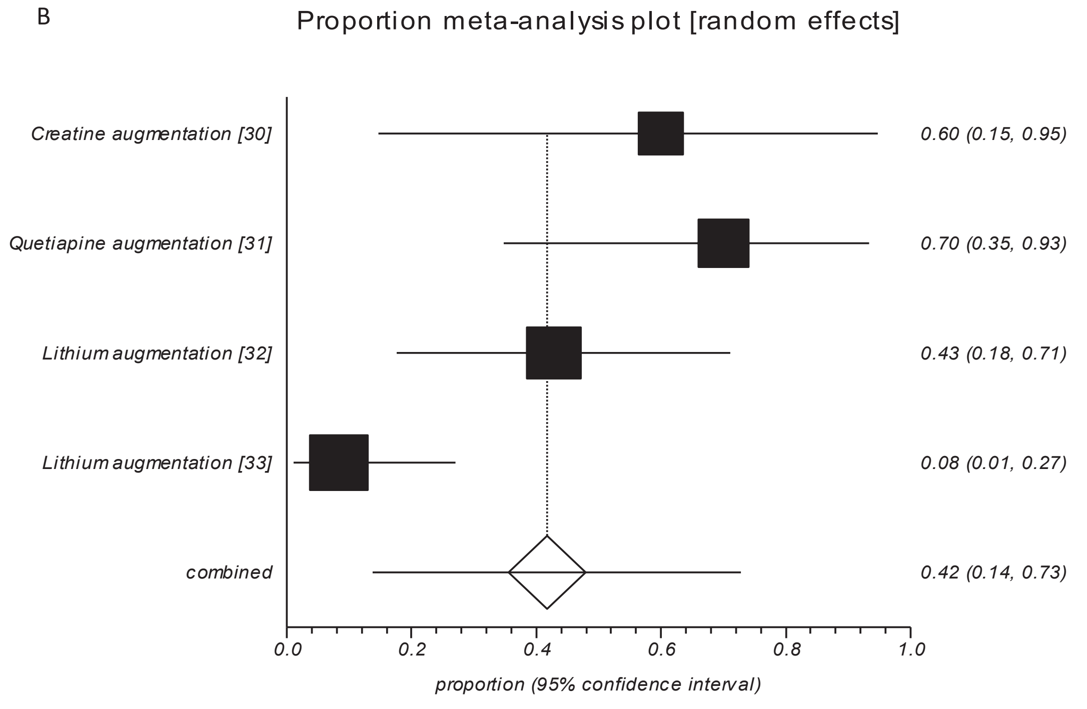

Proportion meta-analysis plot [random effects]
Creatine augmentation [30]
Quetiapine augmentation [31]
Lithium augmentation [32]
Lithium augmentation [33]
combined
0.60 (0.15, 0.95)
0.70 (0.35, 0.93)
0.43 (0.18, 0.71)
0.08 (0.01, 0.27)
0.42 (0.14, 0.73)
0.0
0.2
0.4
0.6
0.8
1.0
proportion (95% confidence interval)
